# Fasting prevents hypoxia-induced defects of proteostasis in *C*. *elegans*

**DOI:** 10.1371/journal.pgen.1008242

**Published:** 2019-06-27

**Authors:** Nicole N. Iranon, Bailey E. Jochim, Dana L. Miller

**Affiliations:** 1 Graduate Program in Molecular and Cellular Biology, University of Washington School of Medicine, Seattle, United States of America; 2 Department of Biochemistry, University of Washington School of Medicine, Seattle, United States of America; Princeton, UNITED STATES

## Abstract

Low oxygen conditions (hypoxia) can impair essential physiological processes and cause cellular damage and death. We have shown that specific hypoxic conditions disrupt protein homeostasis in *C*. *elegans*, leading to protein aggregation and proteotoxicity. Here, we show that nutritional cues regulate this effect of hypoxia on proteostasis. Animals fasted prior to hypoxic exposure develop dramatically fewer polyglutamine protein aggregates compared to their fed counterparts, indicating that the effect of hypoxia is abrogated. Fasting also reduced the hypoxia-induced exaggeration of proteostasis defects in animals that express Aβ_1–42_ and in animals with a temperature-sensitive mutation in *dyn-1*, suggesting that this effect was not specific to polyglutamine proteins. Our data also demonstrate that the nutritional environment experienced at the onset of hypoxia dictates at least some aspects of the physiological response to hypoxia. We further demonstrate that the insulin/IGF-like signaling pathway plays a role in mediating the protective effects of fasting in hypoxia. Animals with mutations in *daf-2*, the *C*. *elegans* insulin-like receptor, display wild-type levels of hypoxia-induced protein aggregation upon exposure to hypoxia when fed, but are not protected by fasting. DAF-2 acts independently of the FOXO transcription factor, DAF-16, to mediate the protective effects of fasting. These results suggest a non-canonical role for the insulin/IGF-like signaling pathway in coordinating the effects of hypoxia and nutritional state on proteostasis.

## Author summary

When blood flow to various parts of the body becomes restricted, those tissues suffer from a lack of oxygen, a condition called hypoxia. Hypoxia can cause cellular damage and death, as in stroke and cardiovascular disease. We have found that in the model organism *C*. *elegans* (a roundworm) specific concentrations of hypoxia cause aggregation of polyglutamine proteins–the same kind of proteins that are found in an aggregated state in the neurodegenerative disorder Huntington’s disease. Here, we show that that worms can be protected from hypoxia-induced protein aggregation if they are fasted (removed from their food source) prior to experiencing hypoxia. Furthermore, we show that the insulin receptor is required for this protection. The insulin receptor is responsible for detecting insulin, a hormone that is released after feeding. Worms with a nonfunctional version of the insulin receptor displayed hypoxia-induced protein aggregation despite being fasted before the hypoxic exposure. Our results highlight a new role for the insulin signaling pathway in coordinating the effects of both hypoxia and nutritional state on protein aggregation.

## Introduction

In order to survive in changing conditions, organisms need to successfully integrate a number of environmental signals and respond appropriately in order to maintain homeostasis. Aerobic heterotrophs must meet their requirements for food and oxygen by taking in these resources from the environment. An inadequate response to low levels of oxygen (hypoxia) can lead to cellular damage or death, an unsurprising outcome given oxygen’s central role in cellular metabolism. Like hypoxia, food deprivation presents an obstacle to homeostasis by impinging on cellular metabolism and disturbing anabolic pathways. However, in many cases food restriction can have beneficial effects, such as extending lifespan and delaying the onset of neurodegenerative diseases and their associated pathologies [1]. In a mouse model of Alzheimer’s disease, 12 weeks of caloric restriction reduces Aβ plaque burden [2], and mice expressing human mutant huntingtin maintained on an alternate-day-feeding diet have reduced brain atrophy and decreased huntingtin aggregate formation [3]. Similarly, depriving *C*. *elegans* of their bacterial food source reduces damage associated with expressing polyglutamine proteins [4].

The protective effect of fasting is not limited to symptoms of neurodegeneration–there are many studies that show fasting can protect against damage associated with hypoxia in mammals. For example, mice on an alternate-day feeding regimen have higher survival rates after myocardial ischemia induced via coronary occlusion [5]. Similar results have been obtained with ischemic damage to the liver. Mice on a calorically restricted diet have reduced infarct damage compared to ad-libitum fed controls [6], and mice that have been fasted for 3 days display reduced hepatocellular apoptosis and damage [7]. Reduced food intake also improves outcomes after cerebral ischemic injury by protecting cortical and striatal neurons [8] and reducing neurological deficits and infarct volume [9]. These observations suggest that understanding the mechanistic basis underlying the protective effects of fasting in hypoxia could provide novel insight into therapeutic strategies to treat pathological conditions associated with ischemia and reperfusion injury.

We have previously shown that in *C*. *elegans* the cellular response to specific hypoxic conditions involves a disruption of proteostasis–the coordination of protein synthesis, folding, degradation, and quality control required to maintain a functional proteome [10]. Here we show that fasting prevents the hypoxia-induced disruption of proteostasis. Our data indicate that the nutritional context of an animal at the onset of hypoxia has the power to alter hypoxia’s effect on proteostasis and that the insulin-like signaling (IIS) pathway plays a role in fasting’s ability to protect against proteostasis decline independently of the canonical downstream transcription factor DAF-16/FOXO.

## Results

In order to investigate the effect of nutritional status on proteostasis in hypoxia, we first used transgenic *C*. *elegans* that express yellow fluorescent protein (YFP) fused to a polyglutamine tract in the body wall muscles [11]. We refer to these animals as QX::YFP, where X refers to the number of glutamine residues fused to YFP, such that Q35::YFP animals express YFP with 35 glutamine residues. In these animals, the number of YFP foci, which correspond to large protein aggregates, can be used as an *in vivo* measure of cellular proteostasis [12].

**Fig 1:**
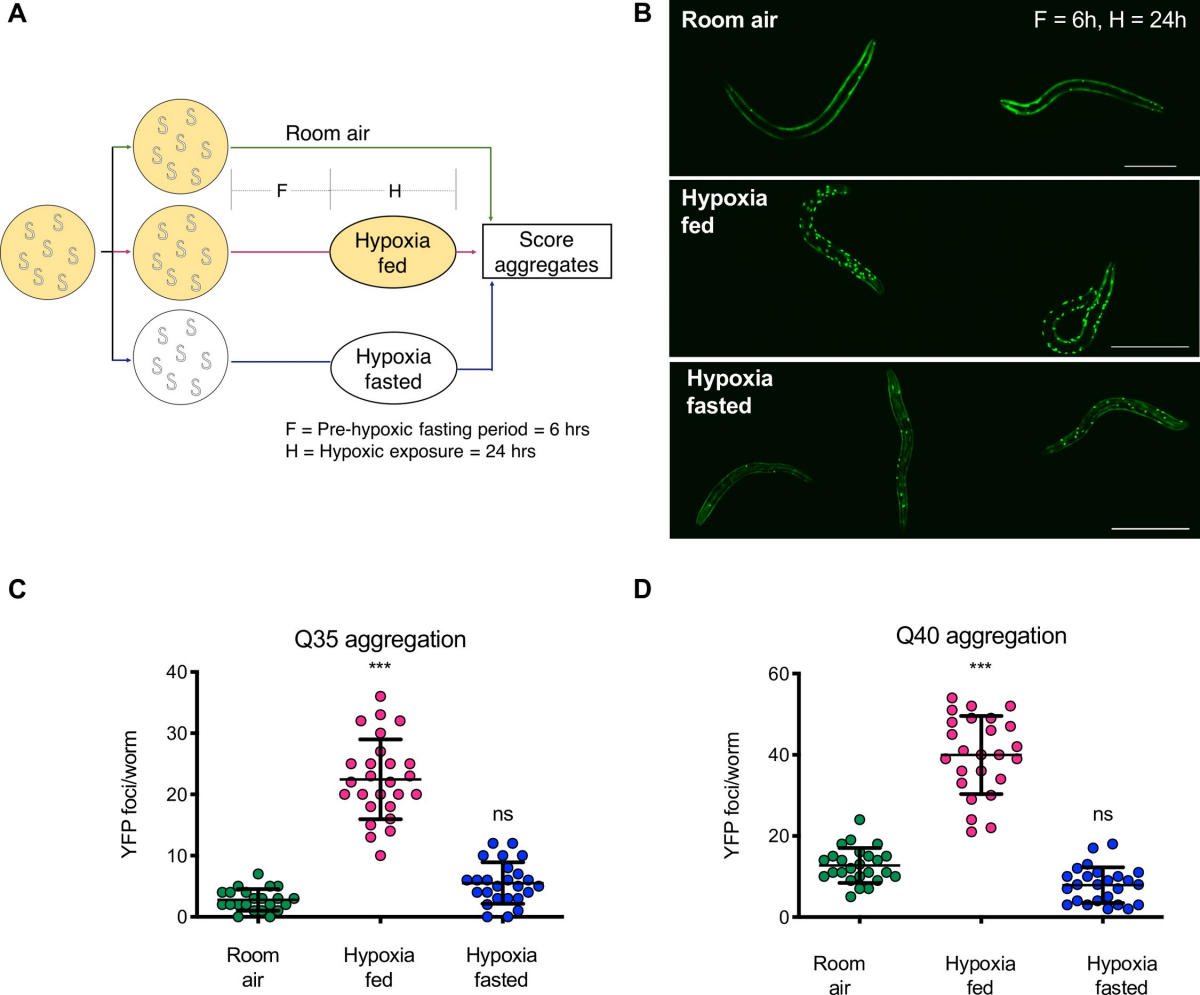
Fasting protects against hypoxia-induced protein aggregation. **A.** Experimental Schematic. Cohorts of age-synchronized animals were split into three groups: the first was maintained on food in room air, the second was maintained on food before and during exposure to hypoxia, and the third was removed from food before exposure to hypoxia. Fasting is indicated by white plates, yellow plates indicate animals on food. F = the duration of fasting (h) before hypoxia; H = duration of hypoxia (h). Unless otherwise noted, aggregates were counted immediately upon removal from hypoxia. **B.** Representative images of Q40::YFP animals from cohorts of animals maintained in room air, exposed to hypoxia on food (hypoxia fed), or exposed to hypoxia while fasted (hypoxia fasted). Note that exposures were not normalized, so protein abundance cannot be estimated from these images. F = 6h, H = 24h. Scale bars = 100μm. **C-D.** Aggregation measurements for L4 Q35::YFP (**C**) and L1 Q40::YFP (**D**) animals exposed to hypoxia on food (fed, magenta) or after removal from food (fasted, blue). Controls remained in room air (green). Data from one representative experiment is shown. Each experiment was repeated at least 3 times, and summary statistics from replicates are in S1 Table. Each circle is the number of YFP foci in a single animal, the mean is indicated by the line, and error bars are the standard deviation. Statistical comparisons were made between animals exposed to hypoxia and controls maintained in room air. Significance: *** *p* < 0.001; ns, not significant.

Exposing animals to 1000 ppm O_2_ (0.1%, with balance N_2_) for 24 hours while fed resulted in an increase in the number of YFP foci (Fig 1B–1D), consistent with previous reports that hypoxia inhibits proteostasis [10]. However, we found that the number of YFP foci that formed in hypoxia was dramatically reduced if the animals were removed from food for six hours before the hypoxic exposure and remained off of food for the duration of hypoxia (Fig 1A). Hypoxia-induced protein aggregation (HIPA) was prevented by fasting in fourth-stage larvae (L4) Q35::YFP animals (Fig 1C) as well as in first-stage larvae (L1) Q40::YFP (Fig 1D). We verified that the bright fluorescent puncta in animals exposed to hypoxia were aggregated protein using fluorescence recovery after photobleaching (S1 Fig), as had been previously observed for age-associated aggregates [11]. We also determined that the abundance of Q35::YFP was the same in animals exposed to hypoxia when fed and fasted (S2 Fig). This is consistent with previous observations that the expression of Q35::YFP did not change even after nine days without food [4]. In control experiments we found no change in the number of protein aggregates between animals in room air that were fed and fasted (S3 Fig). This is likely because we initiated these experiments before much age-associated protein aggregation had occurred, in order to avoid confounding factors from the effects of fasting and hypoxia on aging. From these data, we conclude that fasting prevents HIPA.

We originally chose to fast animals for 6h before exposure to hypoxia to allow animals time to alter gene expression [13]. However, there is no *a priori* evidence that the protective effects of fasting in hypoxia requires changes in gene expression. Therefore, we measured how long of a fasting period was required to mitigate the effects of hypoxia on aggregation of polyglutamine proteins.

**Fig 2:**
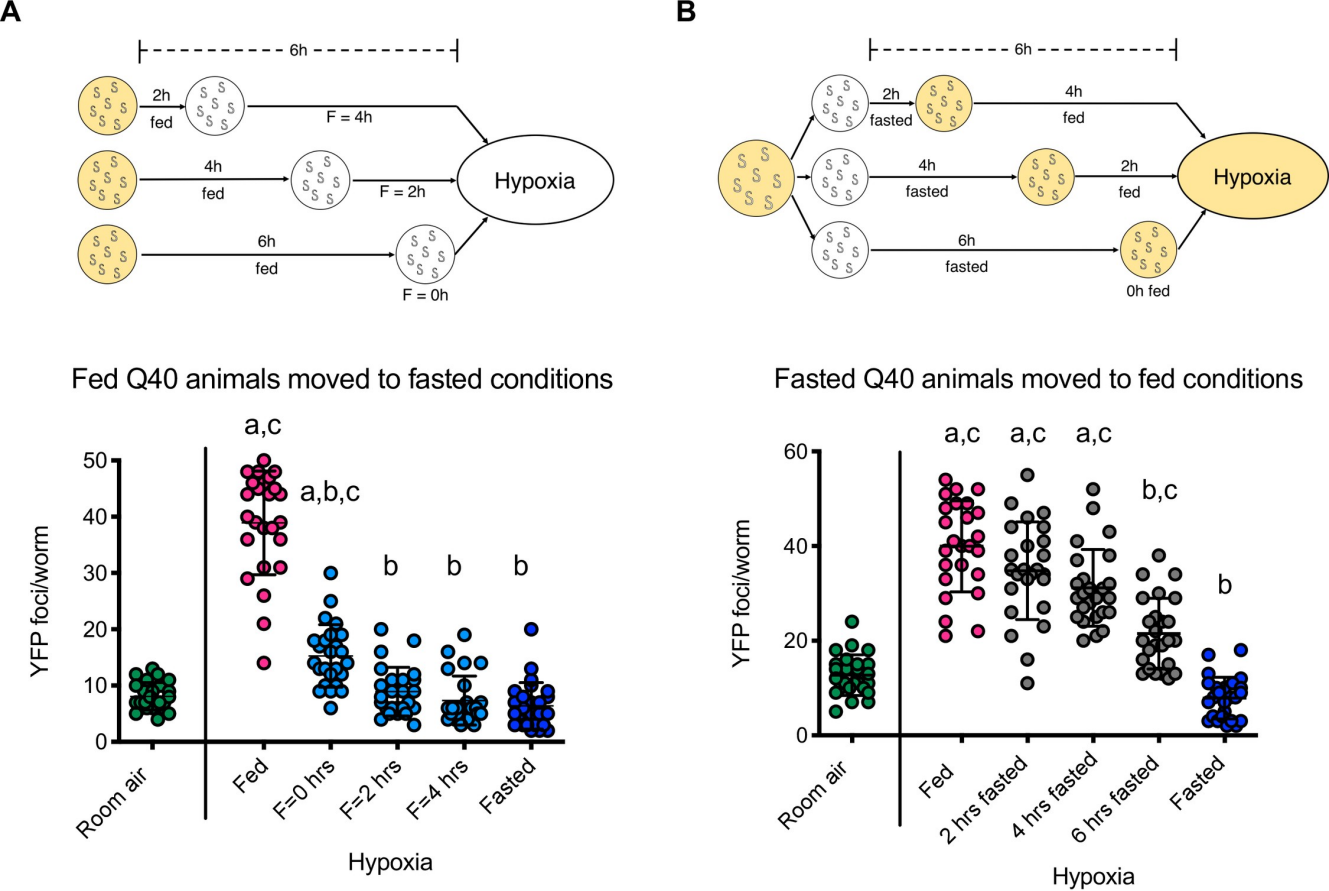
Fasting protection against HIPA is quickly induced and reversed. **A.** Effect of fasting occurs rapidly in hypoxic conditions. Cohorts of L1 Q40::YFP animals were removed from food before exposure to hypoxia (F = 0, 2, or 4 h; H = 24 h). All animals were off of food when exposed to hypoxia and the number of foci was scored immediately upon removal from hypoxia (cyan). Controls remained in room air (green), were continuously on food (fed, magenta), or were fasted for a full 6 h before hypoxia (fasted, blue). Data from one representative experiment is shown. Each circle is the number of YFP foci in a single animal, the mean is indicated by the line, error bars are the standard deviation. Significance was calculated using a Kruskal-Wallis test and Dunn’s multiple comparisons post hoc analysis. Significant differences (*p* < 0.05) in aggregation between conditions are indicated by letters above each group as follows: a—significantly different from room air controls; b—significantly different from fed hypoxic controls; c—significantly different from fasted hypoxic controls. **B.** The protective effects of fasting are rapidly reversed. As shown in the schematic above the graph, cohorts of L1 Q40::YFP animals were removed from food 6h before exposure to hypoxia, and fasted for 2, 4, or 6 h before being returned to food. All cohorts were on food when exposed to hypoxia (H = 24 h). The number of foci was scored immediately upon return to room air (gray). Controls remained in room air (green), were continuously on food and exposed to hypoxia (fed, magenta), or were not returned to food before hypoxia (fasted, blue). Data from one representative experiment is shown. Each circle is the number of YFP foci in a single animal, the mean is indicated by the line, error bars are the standard deviation. Statistical comparisons were made between animals fasted for the indicated amount of time and controls maintained in room air, fed controls exposed to hypoxia after being continuously on food, and fasted controls that were not returned to food before hypoxia. Significance was calculated using a Kruskal-Wallis test and Dunn’s multiple comparisons post hoc analysis. Significant differences (*p* < 0.05) in aggregation between conditions are indicated by letters above each group as follows: a—significantly different from room air controls; b—significantly different from fed hypoxic controls; c—significantly different from fasted hypoxic controls. For all panels, each experiment was repeated at least 3 times, and summary data from replicates are in S2 Table.

To determine the pre-hypoxia fasting duration required to protect against HIPA, we removed Q35::YFP animals from food for varying lengths of time before being exposed to hypoxia (schematic in Fig 2A). We found that animals removed from food immediately before exposure to hypoxia developed significantly fewer YFP foci in hypoxia as compared to controls that remained on food in hypoxia (Fig 2A, 6h fed compared to fed). We conclude that extended fasting before exposure to hypoxia is not required to prevent HIPA. Instead, our data show that the protective effects of fasting occur very rapidly. In fact, the full protection against HIPA is realized with only 2h fasting before exposure to hypoxia (Fig 2A). These results suggest that at least some of the protective effects of fasting do not require a period of adaptation to fasting prior to the hypoxic exposure. Instead, we conclude the environment at the onset of the exposure to hypoxia dictates at least some aspects of the response to hypoxia.

Work in other systems has shown that fasting can have a protective effect that persists even after animals are returned to food [14]. To further explore the requirements for fasting to protect against HIPA we next asked whether the protective effects of fasting against HIPA could be reversed. In these experiments (Fig 2B), we began fasting animals 6h before exposure to hypoxia but then returned the animals to food prior to initiation of hypoxia. We observed that animals fasted for a full 6h and then returned to food immediately before exposure to hypoxia (Fig 2B, 6h fasted) developed significantly more YFP foci than animals that were fasted for 6h and then exposed to hypoxia in the absence of food (Fig 2B, fasted), suggesting that the nutritional context of an animal as it experiences hypoxia is able to mediate the effect of hypoxia on proteostasis. Furthermore, we found no protection from HIPA if animals were fasted for 4h, but then fed for 2 h before exposure to hypoxia (Fig 2B, 4h fasted), even though 4h of fasting was sufficient for complete protection against HIPA in the absence of food (Fig 2A, 2h fed). This result indicates that the protective effects of fasting are fully reversed within 2h of return to food. We conclude that the protective effects of fasting in hypoxia are rapidly reversed.

**Fig 3:**
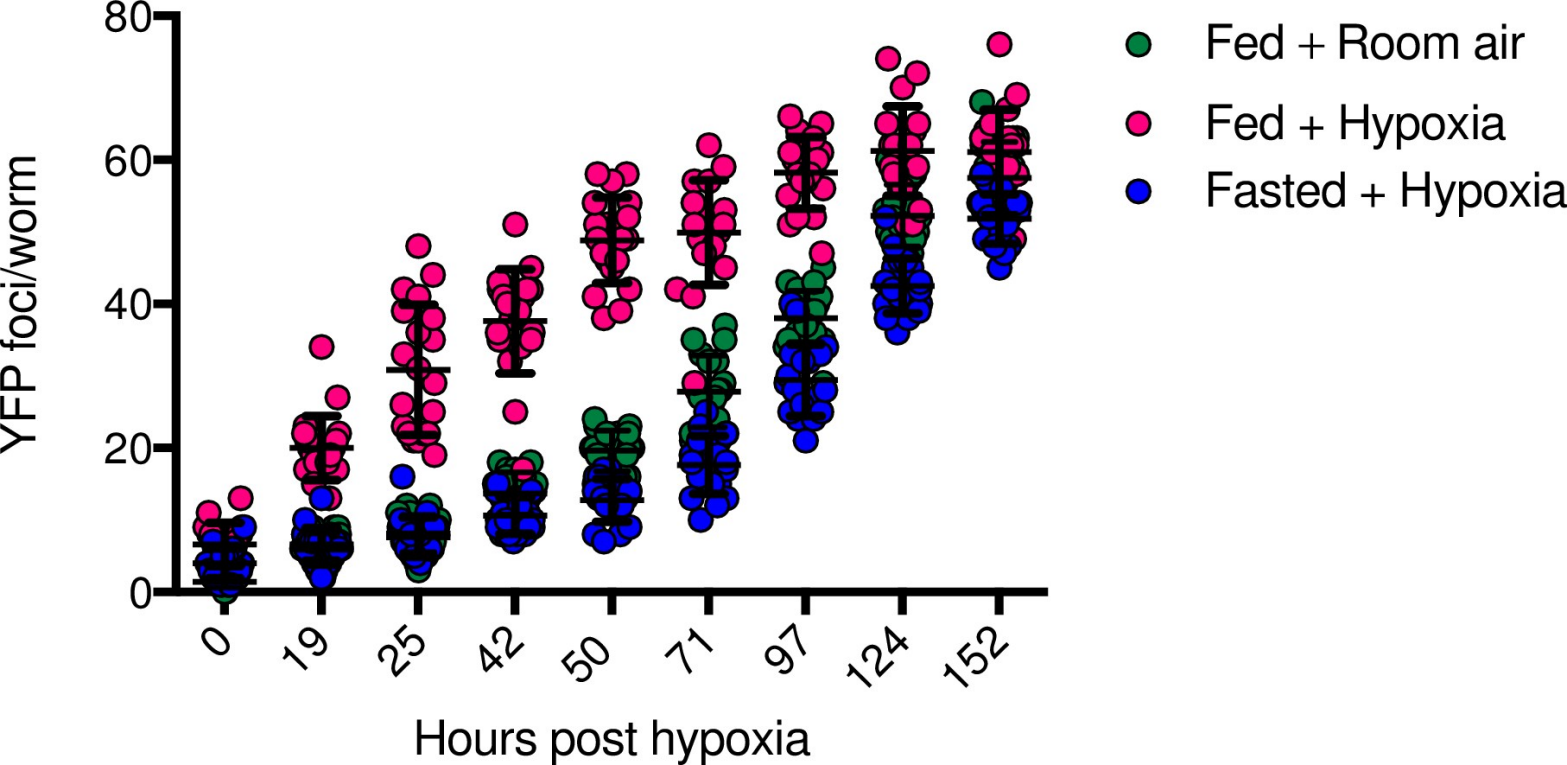
Fasting protects against long-term effects of hypoxia on proteostasis. Cohorts of L4 Q35::YFP animals were exposed to hypoxia (H = 10 h) on food (magenta) or fasted (blue, F = 6h). Controls remained in room air on food (green). The number of YFP foci was scored after return to room air as indicated. Data from one representative experiment is shown. The experiment was repeated at least 3 times. Each cohort included at least 20 animals per time point. Summary statistics from independent replicates are in S10 Table.

Shorter exposures to hypoxia, which do not immediately increase the number of polyglutamine protein aggregates, still disrupt long-term proteostasis as evidenced by the increased rate of age-associated protein aggregation after return to room air [10]. We therefore asked whether fasting could protect against these long-term proteostasis deficits in addition to HIPA. We exposed Q35::YFP L4 animals to hypoxia for only 10h either in the fed state or after fasting for 6h (F = 6 hours, H = 10 hours as per Fig 1A). Control animals remained on food in room air. Immediately after this short hypoxic exposure, there was no observed increase in the number of YFP foci in animals exposed to hypoxia regardless of whether food was present (Fig 3, 0 hours post-hypoxia). As expected, the animals exposed to hypoxia in the fed state accumulate aggregates faster than control animals. In contrast, animals exposed to hypoxia while fasted accumulate YFP foci at the same rate as control animals. Animals that were fasted in room air also accumulated YFP foci at the same rate as room air, fed controls (S3 Fig). These data indicate that fasting both prevents HIPA and protects against the long-term effects on proteostasis induced by a short exposure to hypoxia.

**Fig 4:**
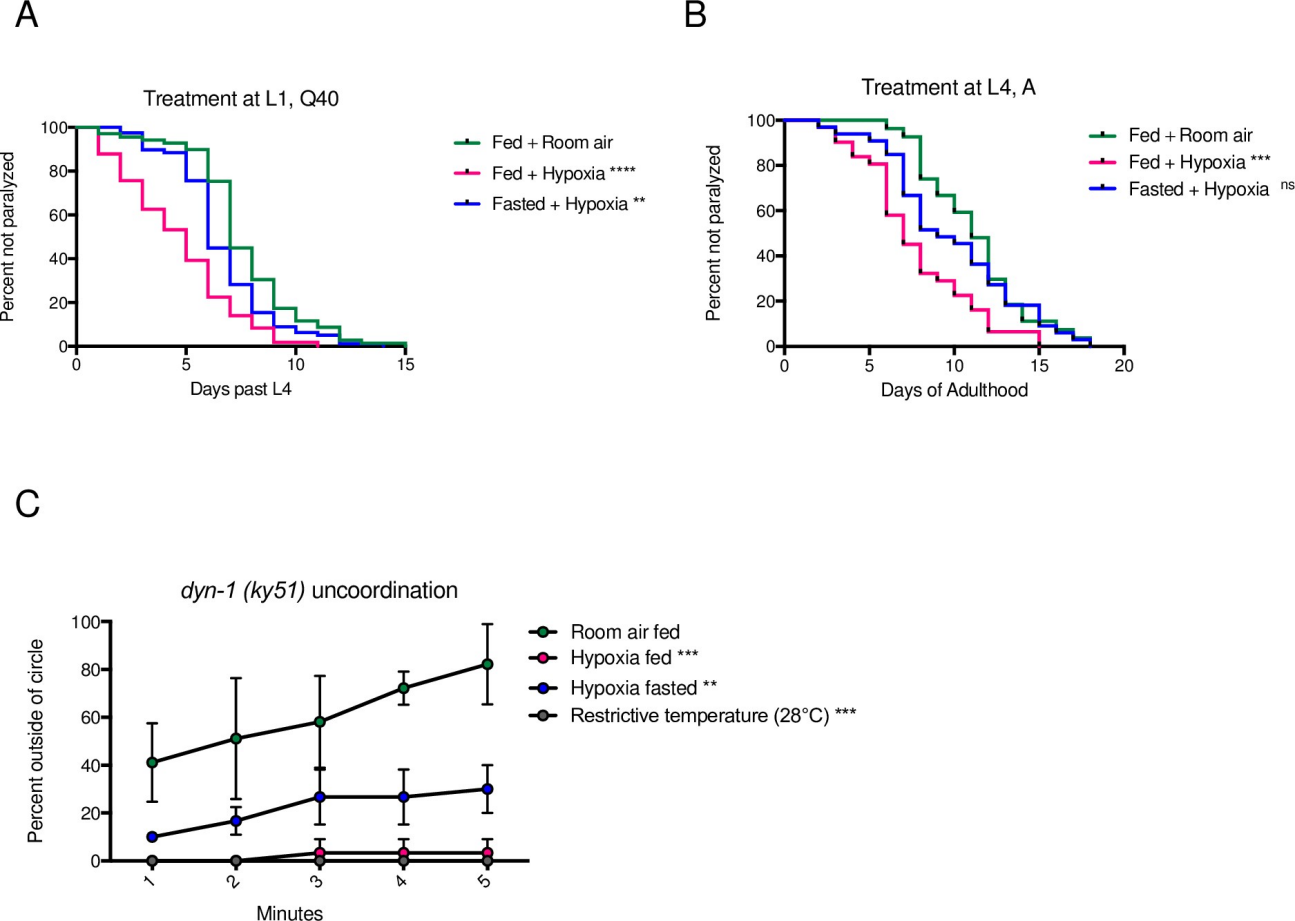
Fasting has general protective effects against hypoxia-induced defects in proteostasis. **A.** Fasting protects against toxicity of Q40::YFP. Cohorts of L1 animals expressing Q40::YFP were exposed to hypoxia on food (magenta), or fasted (blue) before exposure to hypoxia (F = 6h, H = 24 h). Paralysis was scored after return to room air, beginning the first day of adulthood. Controls remained on food in room air (green). Data from one representative experiment is shown, each cohort included at least 70 animals. Each experiment was repeated at least 3 times. Significance was calculated using a Log-rank (Mantel-Cox) test with a Bonferroni correction for multiple comparisons. Statistical comparisons were made between animals exposed to hypoxia and animals maintained in room air. **** *p* < 0.0001; ** *p* < 0.01. **B.** Fasting protects against toxicity of Aβ_1–42_. Cohorts of L4 animals expressing Aβ_1–42_ were exposed to hypoxia on food (magenta) or fasted (blue) before exposure to hypoxia (F = 6h, H = 24h). Paralysis was scored after return to room air, beginning at the first day of adulthood. Controls remained on food in room air (green). Data from one representative experiment is shown, each cohort included at least 70 animals. Each experiment was repeated at least 3 times. Significance was calculated using a Log-rank (Mantel-Cox) test with a Bonferroni correction for multiple comparisons. Statistical comparisons were made between animals exposed to hypoxia and animals maintained in room air. *** *p* < 0.001. **C.** Fasting protects against hypoxia effects on metastable DYN-1. Temperature-sensitive *dyn-1(ky51)* mutant animals were exposed to hypoxia at the permissive temperature on food (magenta), or after fasting (blue). Controls remained on food in room air at the permissive temperature (green) or on food at the non-permissive temperature (28°C, gray). Paralysis was scored 1h after return to room air. Average data from 3 independent experiments is shown, each cohort included 10 animals. Significance was calculated using a repeated measures two-way ANOVA and Dunnett’s multiple comparisons test. Statistical comparisons were made between animals exposed to hypoxia or animals maintained at the restricted temperature and animals maintained in room air. Significance: *** *p* < 0.001; ** *p* < 0.01. Summary statistics for replicates from all experiments are in S4 Table.

The cellular role of protein aggregates is controversial, with some reports finding a protective role and others suggesting a cytotoxic effect [15]. We have previously found that aggregates induced by hypoxia are likely cytotoxic, as they accelerate polyQ-associated paralysis even after animals are returned to room air [10]. We therefore next asked if fasting would protect against increased proteotoxicity in addition to HIPA. To address this, we exposed cohorts of L1 Q40::YFP animals to hypoxia for 24 hours while fed or fasted, then returned the animals to room air and measured the onset of paralysis in each cohort. We found that fasting slowed the rate at which paralysis developed relative to animals exposed to hypoxia while fed (Fig 4A). There was no difference in the rate of paralysis onset if animals were fasted in room air (S3 Fig). This result indicates that fasting protects against hypoxic effects of increased protein aggregation and proteotoxicity.

We next sought to determine whether fasting’s protective effects on proteostasis extend to other models of proteotoxicity. Human amyloid β (Aβ)_1-42_ peptide expressed in the body wall muscles of *C*. *elegans* results in cytoplasmic plaque formation, with a subsequent phenotype of progressive paralysis [16]. *C*. *elegans* expressing Aβ_1–42_ in their body wall muscles become paralyzed more quickly when they are exposed to hypoxia [10]. We found that this effect of hypoxia was reversed by fasting, as the rate that paralysis develops is slowed if animals expressing Aβ_1-42_ are exposed to hypoxia while fasting (Fig 4B). Because Aβ_1–42_ and Q40::YFP are both expressed in body wall muscles, we also evaluated if fasting protected animals expressing a metastable version of the neuronal dynamin protein DYN-1 from the effects of hypoxia. The *dyn-1(ky51)* mutant contains a temperature-sensitive mutation, such that the DYN-1 protein is functional and *dyn-1(ky51)* mutant animals exhibit wild-type motility at the permissive temperature (20°C), but become uncoordinated at the restrictive temperature (28°C) due to improper folding of the DYN-1 protein [17]. Genetic and environmental factors that disrupt proteostasis, including hypoxia, prevent the proper folding of the DYN-1 protein at the permissive temperature, thereby rendering the *dyn-1(ky51)* animals uncoordinated [10,18]. Similar to our experiments with Q40::YFP and Aβ_1–42_, we found that fasting *dyn-1(ky51)* mutant animals before exposure to hypoxia results in a partial rescue of hypoxia-induced uncoordination at the permissive temperature (Fig 4C). Together, our results suggest that fasting has a general protective effect against proteostasis defects induced by hypoxia, and that this protective effect is not specific to a particular tissue, developmental stage, or misfolded/aggregation prone model.

Dysregulation of insulin-like signaling (IIS) has been tied to protein aggregation and neurodegeneration in a number of model organisms [19]. As the IIS pathway links food availability to growth, development, stress resistance, and aging, we hypothesized that changes in IIS could explain how fasting modulates the effect of hypoxia on proteostasis. The IIS pathway is widely conserved in metazoans [20]. We therefore explored the hypothesis that IIS would mediate the effects of fasting to prevent HIPA.

**Fig 5:**
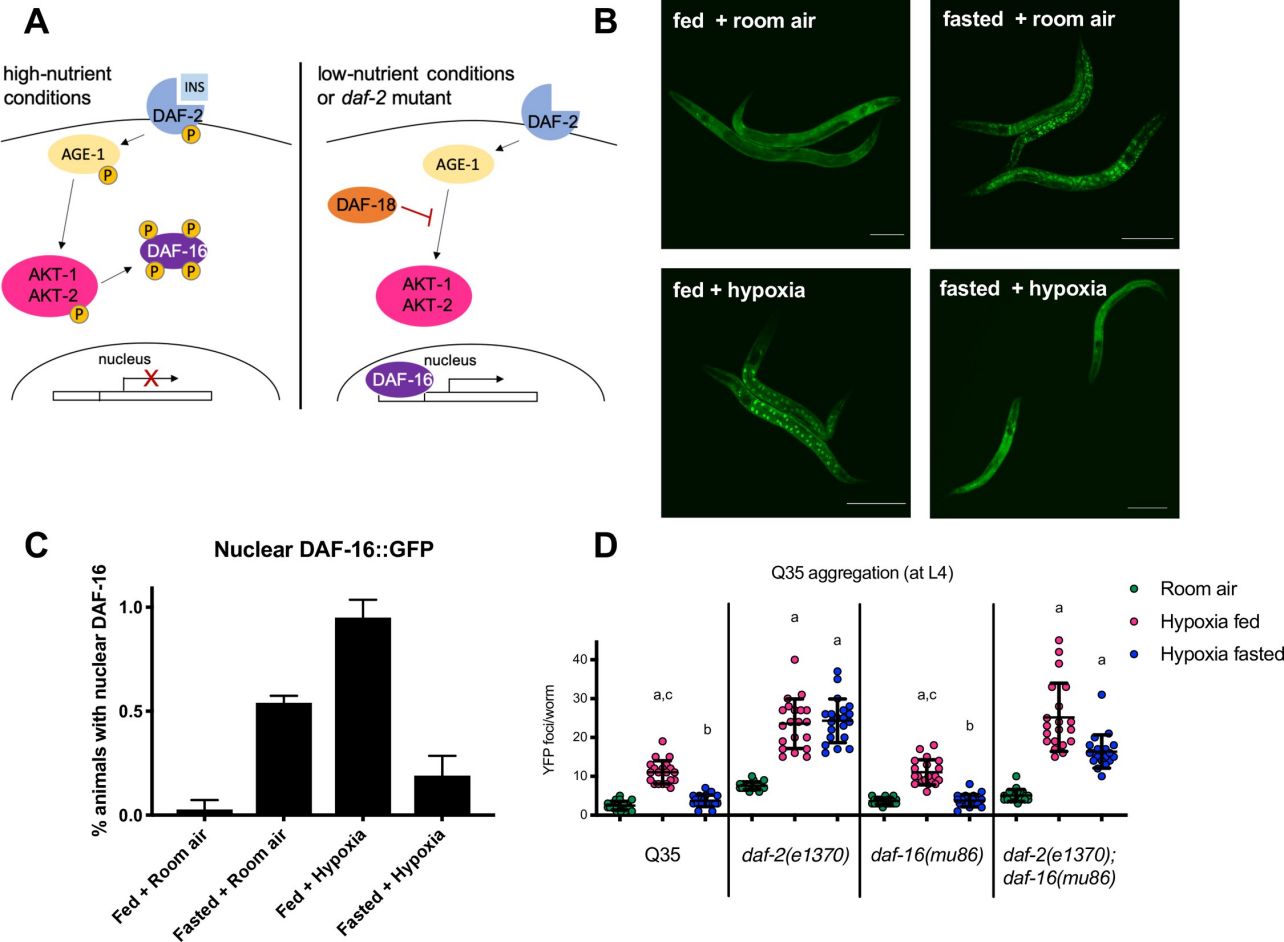
The insulin-like signaling pathway is required for fasting protection. **A.** Schematic of key insulin-signaling pathway members in *C*. *elegans*. Under nutrient-rich conditions, insulin-like peptides bind to the insulin receptor DAF-2, initiating a phosphorylation cascades that ultimately leads to the phosphorylation of the FoxO transcription factor DAF-16, excluding it from the nucleus. Conversely, when nutrients are scarce, DAF-16 remains unphosphorylated and is able to enter the nucleus and bind to its target genes. **B.** DAF-16 is not localized to the nucleus in fasted animals exposed to hypoxia. Cohorts of 20 DAF-16::GFP animals were maintained in room air on food for 24 hrs (fed + room air), fasted in room air for 24 hrs (fasted + room air), exposed to hypoxia for 24 hrs on food (fed + hypoxia), or exposed to hypoxia after fasting (fasted + hypoxia; F = 6h, H = 24hr). Scale bars = 100μm. **C.** Quantification of DAF-16::GFP nuclear accumulation. The percent of animals with nuclear GFP was scored immediately post hypoxia. Average data from 3 independent experiments is shown. The bar height indicates the mean. Error bars are the standard deviation. **D.** Fasting does not protect *daf-2* mutants against HIPA. Aggregation measurements (F = 6h, H = 24h) for L4 Q35::YFP animals with mutations in *daf-2(e1370)*, *daf-16(mu86)*, and the *daf-2(e1370); daf-16(mu86)* double mutant. Animals were maintained on food in room air (room air, green), were exposed to hypoxia on food (fed, magenta), or were exposed to hypoxia after removal from food (hypoxia fasted, blue). Each circle is the number of YFP foci in a single animal, the mean is indicated by the line, error bars are the standard deviation. Data from one representative experiment is shown. Each cohort included at least 20 animals, and each experiment was repeated at least 3 times. Significance was calculated using a Kruskal-Wallis test and Dunn’s multiple comparisons post hoc analysis. Significant differences (*p* < 0.05) in aggregation for a given strain between conditions are indicated by letters above each group as follows: a—significantly different from room air controls; b—significantly different from fed hypoxic controls; c—significantly different from fasted hypoxic controls. Summary statistics from replicate experiments is in S5 Table.

We first looked at the localization of DAF-16::GFP in animals exposed to hypoxia to determine if IIS is active in hypoxia. DAF-16 is the *C*. *elegans* orthologue of the FOXO transcription factor. When active, the insulin/IGF-like receptor DAF-2 initiates a phosphorylation cascade that results in the phosphorylation and nuclear exclusion of DAF-16 protein [21,22]. Conversely, when nutrients are scarce, DAF-16 remains unphosphorylated by upstream kinases and is able to enter the nucleus and bind to its target genes [22,23]. We found that DAF-16::GFP remained diffuse and cytoplasmic in control worms maintained in room air on food (Fig 5B and 5C), but accumulated in the nucleus of animals that were removed from food in room air (Fig 5B and 5C) or were exposed to hypoxia on food (Fig 5B and 5C). These results suggest that IIS activity is reduced by fasting and hypoxia, consistent with previous reports [24,25]. Surprisingly, DAF-16::GFP did not accumulate in the nuclei of animals exposed to hypoxia after fasting (Fig 5B and 5C), despite hypoxia and fasting both individually resulting in nuclear accumulation.

These DAF-16::GFP localization patterns led us to interrogate requirements for DAF-16 and the upstream IIS receptor DAF-2 in mediating fasted and fed responses to hypoxia. To this end, we crossed the Q35::YFP transgene into *daf-2(e1370)* and *daf-16(mu86)* backgrounds. The fact that DAF-16::GFP is localized to the nucleus in fed animals exposed to hypoxia suggests the possibility that DAF-16 facilitates HIPA. However, we found that *Q35*::*YFP; daf-16(mu86)* mutant animals exhibit robust HIPA on food (Fig 5D), indicating that DAF-16 is not required for HIPA despite its nuclear accumulation in fed hypoxic animals. We also asked if there was a genetic requirement for the IIS receptor DAF-2. Our data indicate that IIS does not mediate the effects of hypoxia on proteostasis in fed animals, as *Q35*::*YFP; daf-2(e1370)* mutant animals exhibit robust HIPA when fed (Fig 5D). Thus, neither DAF-16 nor DAF-2 activities are required for HIPA in fed animals.

Given the IIS-independent nature of HIPA in fed animals, we next investigated whether fasting protection requires IIS. We discovered that DAF-2, but not DAF-16 is required for fasting protection against HIPA. Fasting protects the *Q35*::*YFP; daf-16(mu86)* similar to wild-type (Fig 5D); however, we observe significant HIPA when *Q35; daf-2(e1370)* and *Q35; daf-2(e1368)* mutant animals are exposed to hypoxia when fasted (Figs 5D and S4). These results show that protective effects of fasting in hypoxia require DAF-2, but not DAF-16. This is consistent with our observation that DAF-16::GFP is not localized to the nucleus in fasted animals exposed to hypoxia (Fig 5B and 5C).

We found that the insulin/IGF-like receptor DAF-2 mediates the protective effects of fasting on HIPA, while the FOXO transcription factor DAF-16 is not required for protection. Given this finding, we also checked the DAF-16::GFP localization pattern in *daf-2(e1370)* animals. These mutants have constitutively nuclear DAF-16 in the fed state due to decreased signaling through the IIS pathway [22]. Since DAF-16::GFP is not localized to the nucleus in fasting-protected wild-type animals exposed to hypoxia, we sought to investigate whether the nuclear localization of DAF-16 in *daf-2(e1370)* mutants, which are not protected by fasting, would be altered by hypoxia. We found that DAF-16::GFP is fully nuclear in all conditions, including fasted hypoxia, in these animals (S5 Fig).

In *C*. *elegans*, DAF-16 mediates the effects of decreased signaling through DAF-2. Mutations in *daf-16* suppress most *daf-2* mutant phenotypes including increased lifespan, enhanced dauer formation, increased fat storage, reproductive delays, and increased resistance to heat and oxidative stress [26,27]. This coupled with the nuclear localization of DAF-16::GFP in *daf-2* mutants led us to hypothesize that *daf-16* would be required for the HIPA in fasted *Q35; daf-2(e1370)* mutant animals. While *Q35; daf-16(mu86)* mutant animals were protected from HIPA by fasting similar to wild-type controls, *Q35; daf-2(e1370); daf-16(mu86)* animals still exhibit significant HIPA when fasted (Fig 5D). These results indicate that DAF-2 mediates the effects of fasting to prevent HIPA at least partly independently of DAF-16.

We took a candidate approach to attempt to identify factors that act downstream of *daf-2* to protect proteostasis in hypoxia. We focused first on *hif-1*, the hypoxia inducible transcription factor [28]. We previously demonstrated that HIF-1 activity helps to blunt the effects of hypoxia on proteostasis [10]. Moreover, increased lifespan of *C*. *elegans* exposed to hypoxia depends on both *hif-1* and *daf-16* [25]. To determine if *hif-1* is acting downstream of *daf-2* to protect proteostasis in hypoxia we compared *daf-16*; *daf-2*; *Q35*::*YFP* mutant animals with *hif-1*; *daf-16*; *daf-2*; *Q35*::*YFP* animals. We find that deletion of *hif-1* does not suppress increased protein aggregation in fasted animals exposed to hypoxia (S6 Fig). We similarly investigated the role of *skn-1*, which is required for increased lifespan of *daf-2* mutants [29]. Our data show that fasted *skn-1*; *daf-16*; *daf-2*; *Q35*::*YFP* exhibit HIPA that is indistinguishable from the *daf-16*; *daf-2*; *Q35*::*YFP* mutant animals (S6 Fig). Finally, we evaluated whether the heat-shock factor *hsf-1* was involved. HSF-1 is required downstream of DAF-2 for increased lifespan [30]. However, we find that expression of HSF-1 targets *hsp-16*.*2*, *hsp-70*, and *hsp-4* is induced to the same degree in fed and fasted animals exposed to hypoxia (S7 Fig), suggesting that differential activity of HSF-1 does not underlie the protective effects of fasting. In fact, the expression of a variety of genes induces by proteotoxic stress, including the unfolded protein response and autophagy, are similarly induced in fed and fasted animals (S7 Fig). Together, these results suggest that the mechanism(s) by which fasting can protect proteostasis in hypoxia is independent of *daf-16*, *hif-1*, *hsf-1*, and *skn-1*. The components of this *daf-2* dependent pathway that can modulate proteostasis are, as yet, a mystery.

## Discussion

This study illustrates the power of fasting to ameliorate the deleterious effects of hypoxia on proteostasis. These findings are consistent with phenomena that have been observed in mammals–fasting mice for a single day increases survival after kidney ischemia and also reduces ischemic damage to the liver [31]. Our results suggest that the nutritional milieu present at the onset of hypoxia can dictate the effect of hypoxia on proteostasis, as fasting protection against hypoxia can be induced quite quickly. Animals that are removed from food immediately before hypoxia are protected against HIPA to a significant degree, even after being maintained on food for the entire pre-hypoxic period. This implies that worms are integrating information about their environment, including nutrient availability, concurrently with the perception of hypoxia. The importance of the nutritional environment of the animal as it experiences hypoxia is further supported by the fact that we also see a rapid reversal of fasting protection. Worms fasted for six hours but that are moved onto food immediately preceding hypoxia are not as protected against HIPA compared to worms that were fasted and remained off of food for the duration of hypoxia. The speed with which fasting protection can be induced and reversed indicates that protection cannot be explained solely by changes in gene expression resulting in a hypoxia-resistant pre-adapted state. Furthermore, the rapidity with which fasting protection can be reversed suggests that altered gene expression or metabolism resulting from the fasting period is alone insufficient to protect against HIPA. Although *C*. *elegans* enter a reproductive and developmental diapause in 1000 ppm O_2_ [32], the protection conferred by fasting does not represent a simple delay in the onset of proteostasis decline due to the time spent in hypoxia. Rather, fasting provides long-term protection against the accrual of protein aggregates and toxicity even after the return to room air.

We found that the IIS receptor DAF-2 is required for fasting to prevent HIPA. This is somewhat counterintuitive, as decreased function of *daf-2* mutants could be thought of as “phenocopying” the fasted situation. Consistent with this, both fasting and mutation of *daf-2* lead to increased nuclear localization of DAF-16. However, our results show that *daf-2* mutant animals do not phenocopy wild-type, fasted animal (which show little HIPA). In contrast, *daf-2* mutant animals exhibit robust HIPA regardless of whether they are fed or fasted. Moreover, we found that while hypoxia and fasting individually promote the nuclear localization of DAF-16::GFP, there is no nuclear accumulation in in fasted animals exposed to hypoxia. These results suggest that activation of DAF-2 in fasted animals is required to prevent hypoxia-induced perturbations of proteostasis.

Although required for the protective effects of fasting in hypoxia, our data show that IIS is not required for the normal response to hypoxia in fed animals–both *daf-16*/FOXO and *daf-2*/IR mutants have relatively normal HIPA when fed. This contrasts with previous studies that show *C*. *elegans daf-2* mutant animals are resistant to anoxia, displaying reduced muscle and neuronal cell death following anoxia [33,34]. Similarly, flies with defective insulin signaling due to mutations in the insulin receptor *InR*, or *Chico*, the insulin receptor substrate, are protected against anoxia/reoxygenation injury [35]. The discordance between our results and these previous studies may be due to the fact that the phenotypic and genetic responses to hypoxia depend strongly on the precise concentration of O_2_ available (reviewed in [36]); in our studies we focused on hypoxic conditions with 1000 ppm O_2_, whereas the anoxic conditions used in these previous studies had far less O_2_ available. Our results suggest that, in fed animals, IIS is not required for nor can it protect against hypoxia-induced disruption of proteostasis. Mammalian systems offer precedents of insulin receptor mutations causing sensitivity to hypoxic stress. Knockdown of neuronal insulin-like growth factor 1 receptor (IGF-1R) exacerbates hypoxic injury and increases mortality in mice [37], and IGF-1R is required in order for IGF-1 to protect myocardial cell exposed to ischemia [38]. However, data on the role of mammalian IIS in response to hypoxia are mixed, and are complicated by the fact that different types of insulin receptors mediate distinct cellular functions [39]. As such, the simplified *C*. *elegans* IIS system may be useful for understanding contextual inputs that alter IIS outputs.

DAF-16 is believed to be the main nexus of IIS [22,30,40,41], which makes the DAF-2-dependent, but DAF-16-independent nature of the protective effect of fasting we have described unusual in *C*. *elegans*. Decreased DAF-2 activity results in phenotypes such as increased lifespan, reproductive delays, and increased resistance to heat and oxidative stress, all of which require DAF-16 [27]. However, a few other examples exist in the literature of DAF-2 dependent, DAF-16 independent phenomena: dauer formation at 27°, meiotic progression of oocytes, salt chemotaxis learning, and regulation of the *dao-3* and *hsp-90* genes [40,42–45]. Our studies suggest that fasting-mediated protection against HIPA is mediated by factors that act downstream of DAF-2, but separate from DAF-16. Understanding the nature of these factors could reveal new aspects of how IIS modulates stress responses and proteostasis in animals.

## Materials and methods

### *C*. *elegans* strains and methods

Animals were maintained on nematode growth media (NGM) with OP50 *E*. *coli* at 20°C [46]. See S10 Table for worm strains. Strains were obtained from the *Caenhorabditis* Genetics Center at the University of Minnesota. Double and triple mutants were generated using standard genetic techniques, and genotypes were verified using PCR.

### Construction of hypoxic chambers

Hypoxic conditions were maintained using continuous flow chambers, as described in [49]. Compressed gas tanks (1000 ppm O_2_ balanced with N_2_) were Certified Standard (within 2% of target concentration) from Airgas (Seattle, WA). Oxygen flow was regulating using Aalborg rotameters (Aalborg Intruments and Controls, Inc., Orangeburg, NY, USA). Hypoxic chambers (and room air controls) were maintained in a 20°C incubator for the duration of the experiments.

### YFP::polyQ aggregation assays

Synchronous cohorts of L1 YFP::polyQ_40_ animals were generated by either bleaching first-day adult animals in a 20% alkaline hypochlorite solution or allowing first-day adult animals to lay eggs for 1–2 hrs on seeded NGM plates. The adults were then removed, and the plates were incubated at 20°C. The next morning, cohorts of hatched L1 larvae were suspended in M9 and mouth-pipetted to new NGM plates for hypoxic exposure. Synchronous cohorts of L4 YFP::polyQ_35_ animals were generated by picking L4 animals from well-fed, logarithmically growing populations.

Cohorts of 25–35 YFP::polyQ animals were exposed to hypoxia for approximately 24 h at 20°C on unseeded 3 cm NGM plates with 40mg/mL carbenicillin or NGM plates seeded with live OP50 food. Plates were ringed with palmitic acid (10mg/mL in ethanol), creating a physical barrier around the edge of each plate to discourage animals from leaving the surface of the agar.

To quantify the number of YFP foci, worms were mounted a 2% agar pad in a drop of 50mM sodium azide as anesthetic. Control experiments showed that azide did not affect the aggregation of YFP::polyQ_35_ or YFP::polyQ4_0_ [47]. YFP foci were identified and quantified as described in [11] and [48]. A Nikon 90i fluorescence microscope with the YFP filter and 10x objective (Nikon Instruments Inc., Melville, NY, USA) was used to visualize and quantify aggregates. In all experiments, the number of aggregates was counted blind to treatment and genotype. Statistical significance was evaluated by calculating P-values between conditions using a Kruskal-Wallis test and Dunn’s multiple comparisons post hoc analysis in GraphPad Prism version 7.0c for Mac OSX (GraphPad Softare, San Diego, California, USA) In all cases, P < 0.05 was considered statistically significant.

### Paralysis and uncoordination assays of proteotoxicity

Animals expressing Aβ_1–42_ or YFP::polyQ_40_ were exposed to 1000 ppm O_2_ for 24 at 20°C as L4 or L1, respectively. For both, animals were grown on seeded NGM plates until 6 hrs before hypoxic exposure, at which point fasted animals were transferred to unseeded NGM plates, where they remained until the end of the hypoxic exposure. Fed animals were transferred to new seeded NGM plates. After hypoxic exposure, all animals were returned to food and normoxia, and incubated at 20°C. Paralysis was scored daily. Worms were considered paralyzed if they failed to respond, other than with movement of the nose or pharyngeal pumping, when tapped with a platinum wire pick 3 consecutive times. Dead or bagged worms were censored from the experiment on the day of death/bagging. Paralyzed worms were removed from the plate on the day of paralysis. Live worms that were not paralyzed were moved to a new plate each day until all worms were scored as either paralyzed or dead. Statistical significance was calculated using Kaplan-Meier log-rank (Mantel-Cox) tests and a Bonferroni correction for multiple comparisons using GraphPad Prism version 7.0c for Mac OSX (GraphPad Softare, San Diego, California, USA).

### DAF-16::GFP localization

Synchronous cohorts of L2 animals expressing DAF-16::GFP were exposed to hypoxia for 24 h at 20°C on unseeded 3 cm NGM plates with 40mg/mL carbenicillin or NGM plates seeded with live OP50 food. Plates were ringed with palmitic acid (10mg/mL in ethanol), creating a physical barrier around the edge of each plate to discourage animals from leaving the surface of the agar. To visualize the localization of DAF-16::GFP, worms were mounted a 2% agar pad in a drop of 10mM levamisole as anesthetic. A Nikon 90i fluorescence microscope with the GFP filter and 10x objective (Nikon Instruments Inc., Melville, NY, USA) was used to visualize DAF-16::GFP. For quantification, percent of animals with nuclear GFP was scored immediately after removal from hypoxia. In all experiments, the GFP localization was scored blind to treatment and genotype. Statistical significance was evaluated by calculating P-values between conditions using a Kruskal-Wallis test and Dunn’s multiple comparisons post hoc analysis in GraphPad Prism version 7.0c for Mac OSX (GraphPad Softare, San Diego, California, USA). P < 0.05 was considered statistically significant.

### qRT-PCR

Animals were grown on NGM plates seeded with OP50 *E*. *coli* at 20°C. When animals reached gravid adult, synchronized embryos were obtained by a 5-minute bleach in 1:1:5 water:KOH:hypochloric acid solution. For each strain/condition, ~9,000 embryos were plated onto a 150 mM NGM plate seeded with live OP50 *E*. *coli*. Animals were not allowed to starve out the plate at any time during the experiment. When animals reached L4, they were exposed to hypoxia with or without a 6 hour fasting period, or were left in room air as controls. Animals were harvested into 1 mL Trizol solution and immediately frozen in liquid nitrogen, as described previously [49]. RNA was isolated from the Trizol preparation as described previously [50]. cDNA was made using Invitrogen SuperScript III First Strand Synthesis System. qPCR was performed using Kappa SYBR FAST qPCR Kit. PCR cycle was as follows: 95C for 3 min, 95C for 15 sec, 55C for 15 sec x40. 4°C to hold. qRT-PCR values were analyzed as described in [51]. In summary, ΔCt for each gene product was calculated by subtracting Ct values from the geometric mean of the control targets that are not altered in response to fasting or hypoxia (*hil-1*, *irs-2*, and *tba-1*). ΔCt were averaged across experiments. Student’s t-test was used to evaluate differences between ΔCt values of treated samples and untreated controls. For differences between genotypes, p-values were calculated with a one-way ANOVA from summary statistics (mean, standard error, n). Reported fold-changes were calculated as 2^-ΔΔCt where ΔΔCt = ΔCt(experimental condition)—ΔCt(control condition). Error bars on graphs represent standard error of the mean.

## Supporting information

S1 Fig Fluorescence recovery after photobleaching (FRAP) of hypoxia-induced protein Q35::YFP foci. Relative fluorescence intensity after photobleaching was quantified for diffuse Q35::YFP in the body wall muscles (orange). Measurements were taken every 30s after bleaching. The diffuse GFP sample (orange) recovered completely before the first measurement after bleaching. In contrast, fluorescence recovery for foci formed in hypoxia both in fed (magenta) and fasted (blue) animals did not recover after photobleaching. Animals were in 1000 ppm O_2_ for 24 h, and fasted animals were removed from food 6h prior to hypoxic exposure. This was also observed for foci that form with age in animals that remained in room air (green), which is consistent with previous studies [11]. Data shown are the average of 5 independent experiments, with the error bars indicating the standard deviation. Numerical data are included in S9 Table.
(TIF)

S2 Fig Fasting does not reduce polyQ::YFP protein abundance. Representative Western blot showing signal from α-GFP antibody, which also recognizes YFP. Animals expressing Q35::YFP were harvested after each treatment into protein loading buffer with SDS and DTT, and boiled. Samples were run on SDS-PAGE and Western blot for actin was used to normalize all samples for protein content. Then, because Q35::YFP expression is so high, samples were diluted and run for α-GFP Western blot in order to ensure that the signal was within the dynamic range of detection. Dilutions are indicated above each graph. α-GFP signals were normalized to room air, fed control samples. Data from three independent replicates are included (filled circles). There is no significant difference in the intensity of bands from animals exposed to hypoxia when fed (magenta bars) or fasted (blue bars). GFP levels in animals fasted in room air are in orange.
(TIF)

S3 Fig Room air fasting does not change aggregation of polyQ proteins. **A.** Fasting in room air does not change Q40::YFP aggregation. L1 animals were exposed to hypoxia (1000 ppm O_2_) for 24 h on plates with or without food. The number of YFP foci was scored immediately upon return to room air. ns, not significantly different than room air fed controls (p > 0.05); * significantly different than room air fed controls. **B.** Fasting in room air does not change aggregation of Q35::YFP. Q35::YFP animals were moved to plates with food (fed, green) or without food (fasted, orange), for 24h and then returned to plates with food. The number of YFP foci was measured 24 and 48 hours later. **C.** Fasting in room air does not change polyglutamine-associated paralysis. Q40::YFP animals were starved for 24h as L1, then returned to food. Paralysis was measured starting at day 1 adult as in Fig 4. Summary statistics from independent replicates for all panels are in S6 Table.
(TIF)

S4 Fig Fasting does not protect *daf-2(e1368)* mutants against HIPA. Aggregation measurements (F = 6h, H = 24h) for L4 *daf-2(e1368)* Q35::YFP animals. Animals were maintained on food in room air (green), were exposed to hypoxia on food (magenta), or were exposed to hypoxia after removal of food (blue). Each circle is the number of YFP foci in a single animal. The mean in indicated by the line, error bars are the standard deviation. Data from one representative experiment is shown. Each cohort included at least 20 animals, and the experiment was repeated at least 3 times. Significance was calculated using a Kruskal-Wallis test and Dunn’s multiple comparisons post hoc analysis. Significant differences (p<0.05) in aggregation for a given strain between conditions are indicated by letters above each group as follows: a—significantly different from room air controls; b -significantly different from fed hypoxic controls; c—significantly different from fasted controls. Summary statistics from independent replicates are in S7 Table.
(TIF)

S5 Fig DAF-16::GFP in *daf-2(e1370)* mutants is localized to the nucleus in all fasted, fed, and hypoxic conditions. Cohorts of 20 *daf-2(e1370)* mutants expressing DAF-16::GFP were maintained in room air on food for 24 hours (Fed + Room air), fasted in room air for 24 hours (Fasted + Room air), exposed to hypoxia for 24 hours on food (Fed + Hypoxia), or exposed to hypoxia after fasting (Fasted + Hypoxia; F = 6h; H = 24H). The percent of animals with nuclear GFP was scored immediately post hypoxia. Average data from 3 independent experiments is shown. The bar height indicates the mean. Error bars (present, but not visible) are the standard deviation.
(TIF)

S6 Fig *hif-1* and *skn-1* do not act downstream of *daf-2* to mediate protective effects of fasting on protein homeostasis. The number of Q35::YFP foci formed after exposure to hypoxia was determined for *hif-1(ia04); daf-16(mu86); daf-2(e1370); rmIs132* triple mutant animals, and for *skn-1(zu169); daf-16(mu86); daf-2(e1370); rmIs132* triple mutants. Animals were exposed to hypoxia for 24h and the number of YFP foci was scored immediately, as in Fig 5. Data from one representative experiment are shown. Each cohort included at least 20 animals, and each experiment was repeated at least 3 times. Significance was calculated using a Kruskal-Wallis test and Dunn’s multiple comparisons post hoc analysis. Significant differences (p < 0.05) in aggregation for a given strain between conditions are indicated by letters above each group as follows: a—significantly different from room air controls; b—significantly different from fed hypoxic controls; c—significantly different from fasted hypoxic controls. Summary statistics from independent replicates are in S16 Table.
(TIF)

S7 Fig Expression of stress response genes, including *hsf-1* targets, is induced by exposure to hypoxia similarly in fed and fasted animals. qRT-PCR measurement of mRNA abundance for each gene was measured from three independent replicates. The *acs-2* and *fat-3* genes are known to by induced by fasting [13]. *phy-2* induction in hypoxia is *hif-1*-dependent [S52]. HSF-1 mediates expression of *hsp-16*.*2*, *hsp-70*, and *hsp-4* [S53–55]. There was no statistically-significant difference between expression of any of these genes in animals exposed to hypoxia when fed or fasted.
(TIF)

S1 Table Summary statistics for replicate experiments for Fig 1. (XLSX)

S2 Table Summary statistics for replicate experiments for Fig 2. (XLSX)

S3 Table Summary statistics for replicate experiments for Fig 3. (XLSX)

S4 Table Summary statistics for replicate experiments for Fig 4. (XLSX)

S5 Table Summary statistics for replicate experiments for Fig 5. (XLSX)

S6 Table Summary statistics for replicate experiments for S3 Fig. (XLSX)

S7 Table Summary statistics for replicate experiments for S4 Fig. (XLSX)

S8 Table Summary statistics for replicate experiments for S6 Fig. (XLSX)

S9 Table Numerical data for FRAP experiments in S1 Fig. (XLSX)

S10 Table *C*. *elegans* strains used in this study. (DOCX)

S1 Text Supplemental references. (DOCX)
