# Supplementary figures and images for: Fasting prevents hypoxia-induced defects of proteostasis in C. elegans
Source: PLoS Genet. 2019 Jun 27;15(6):e1008242. doi: 10.1371/journal.pgen.1008242 (PMC6619831; doi:10.1371/journal.pgen.1008242)

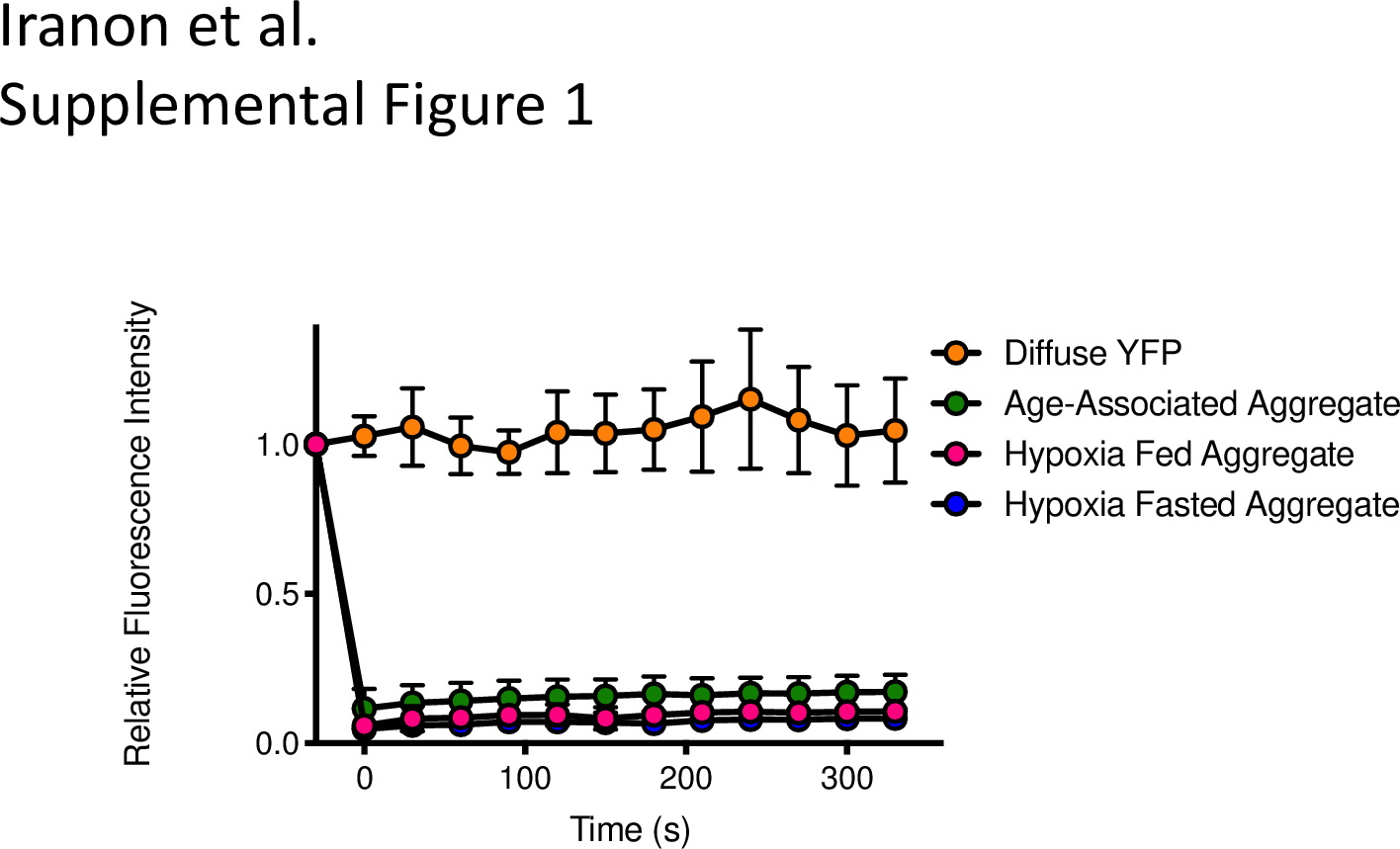

Supplement: S1 Fig — Relative fluorescence intensity after photobleaching was quantified for diffuse Q35::YFP in the body wall muscles (orange). Measurements were taken every 30s after bleaching. The diffuse GFP sample (orange) recovered completely before the first measurement after bleaching. In contrast, fluorescence recovery for foci formed in hypoxia both in fed (magenta) and fasted (blue) animals did not recover after photobleaching. Animals were in 1000 ppm O2 for 24 h, and fasted animals were removed from food 6h prior to hypoxic exposure. This was also observed for foci that form with age in animals that remained in room air (green), which is consistent with previous studies [11]. Data shown are the average of 5 independent experiments, with the error bars indicating the standard deviation. Numerical data are included in S9 Table. (TIF) [file pgen.1008242.s001.tif]

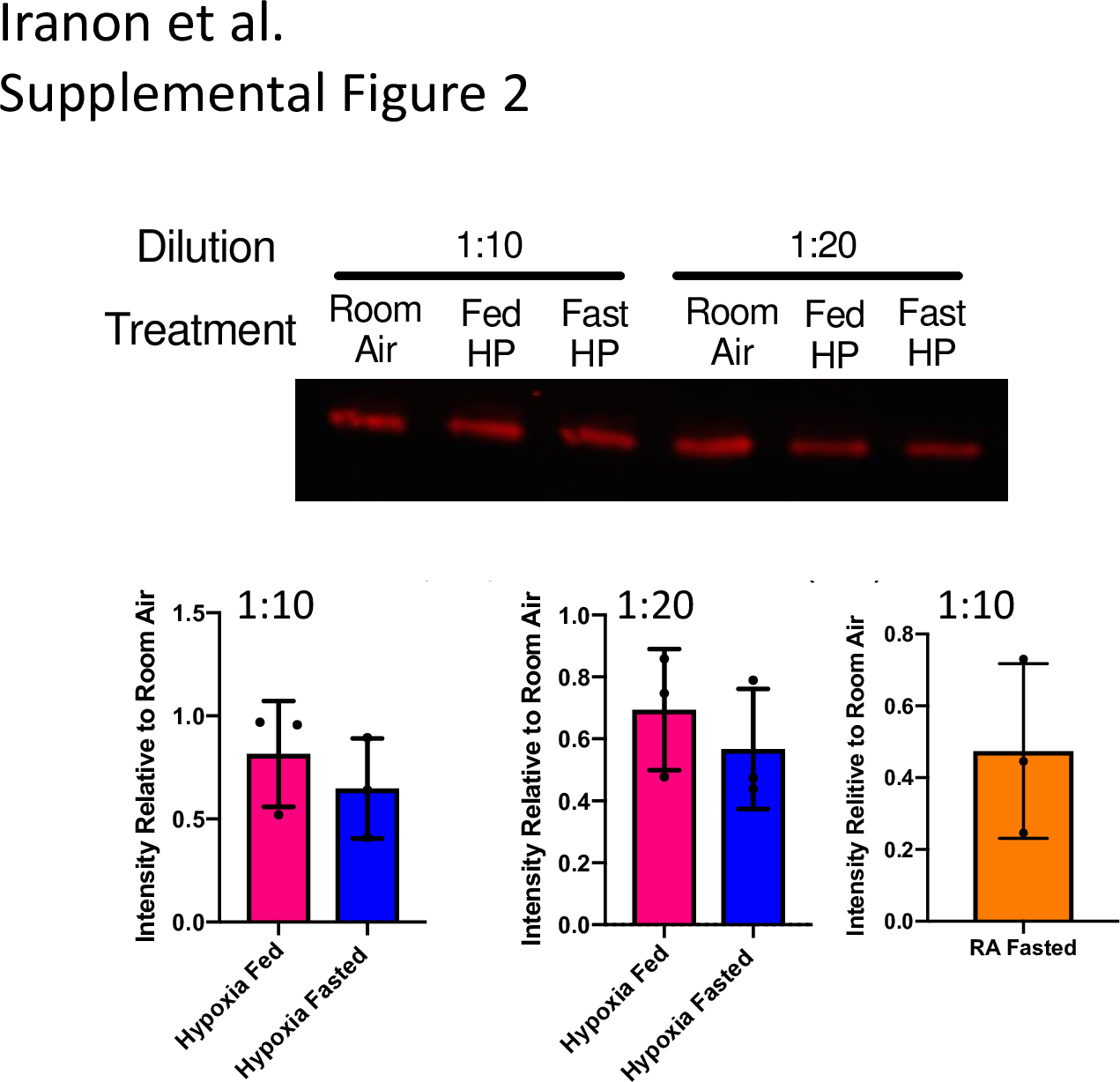

Supplement: S2 Fig — Representative Western blot showing signal from α-GFP antibody, which also recognizes YFP. Animals expressing Q35::YFP were harvested after each treatment into protein loading buffer with SDS and DTT, and boiled. Samples were run on SDS-PAGE and Western blot for actin was used to normalize all samples for protein content. Then, because Q35::YFP expression is so high, samples were diluted and run for α-GFP Western blot in order to ensure that the signal was within the dynamic range of detection. Dilutions are indicated above each graph. α-GFP signals were normalized to room air, fed control samples. Data from three independent replicates are included (filled circles). There is no significant difference in the intensity of bands from animals exposed to hypoxia when fed (magenta bars) or fasted (blue bars). GFP levels in animals fasted in room air are in orange. (TIF) [file pgen.1008242.s002.tif]

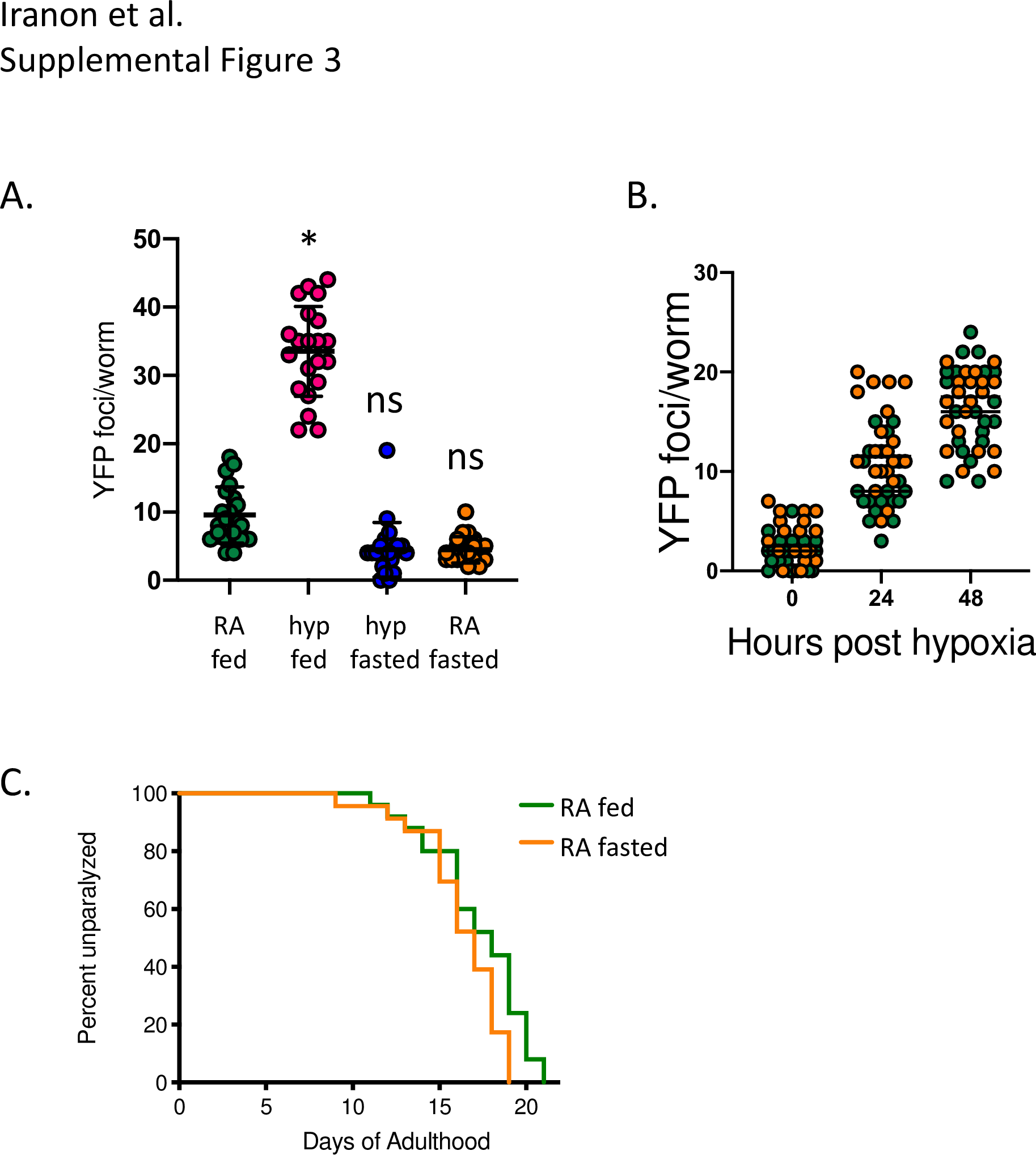

Supplement: S3 Fig — A. Fasting in room air does not change Q40::YFP aggregation. L1 animals were exposed to hypoxia (1000 ppm O2) for 24 h on plates with or without food. The number of YFP foci was scored immediately upon return to room air. ns, not significantly different than room air fed controls (p > 0.05); * significantly different than room air fed controls. B. Fasting in room air does not change aggregation of Q35::YFP. Q35::YFP animals were moved to plates with food (fed, green) or without food (fasted, orange), for 24h and then returned to plates with food. The number of YFP foci was measured 24 and 48 hours later. C. Fasting in room air does not change polyglutamine-associated paralysis. Q40::YFP animals were starved for 24h as L1, then returned to food. Paralysis was measured starting at day 1 adult as in Fig 4. Summary statistics from independent replicates for all panels are in S6 Table. (TIF) [file pgen.1008242.s003.tif]

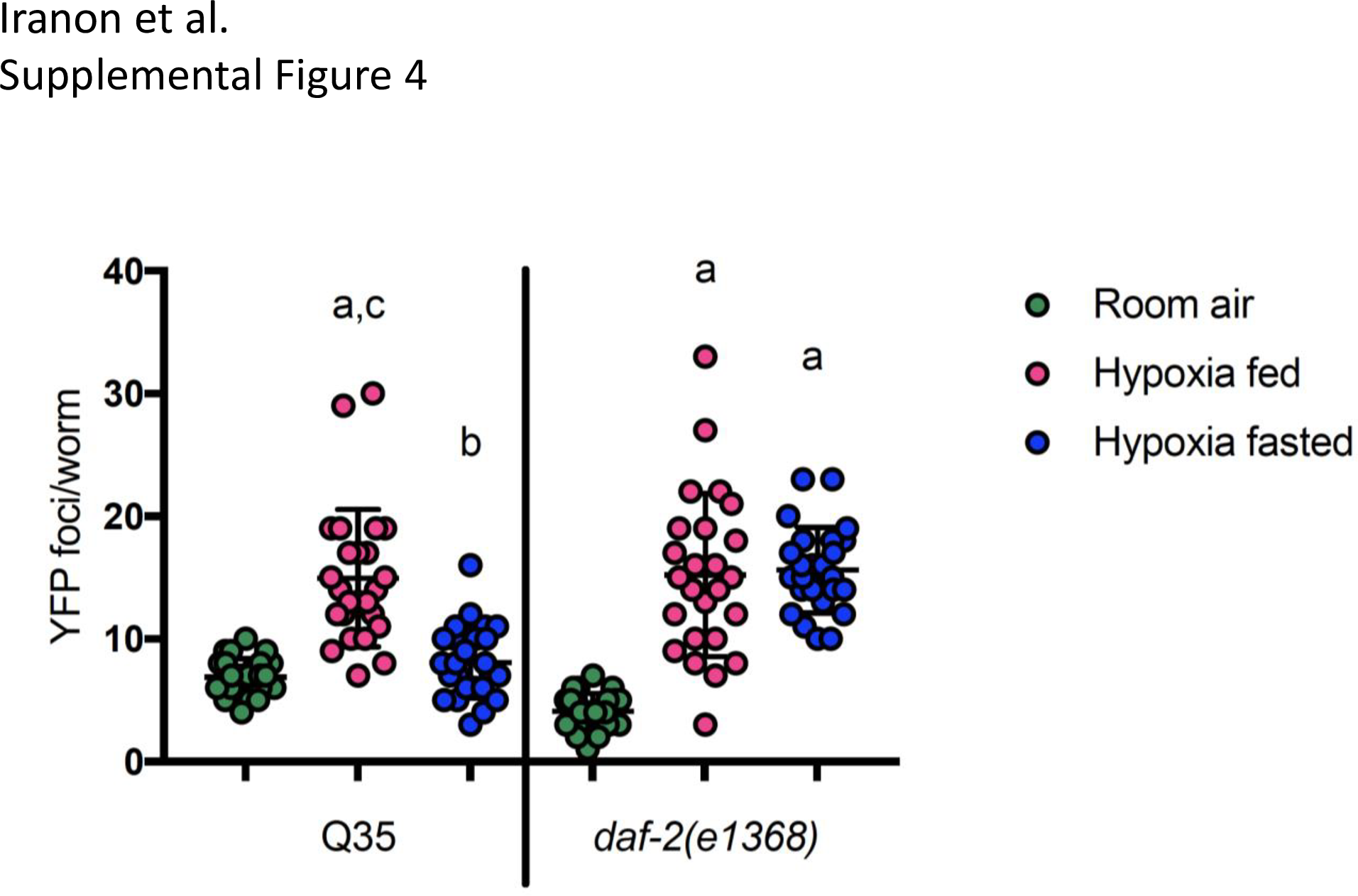

Supplement: S4 Fig — Aggregation measurements (F = 6h, H = 24h) for L4 daf-2(e1368) Q35::YFP animals. Animals were maintained on food in room air (green), were exposed to hypoxia on food (magenta), or were exposed to hypoxia after removal of food (blue). Each circle is the number of YFP foci in a single animal. The mean in indicated by the line, error bars are the standard deviation. Data from one representative experiment is shown. Each cohort included at least 20 animals, and the experiment was repeated at least 3 times. Significance was calculated using a Kruskal-Wallis test and Dunn’s multiple comparisons post hoc analysis. Significant differences (p<0.05) in aggregation for a given strain between conditions are indicated by letters above each group as follows: a—significantly different from room air controls; b -significantly different from fed hypoxic controls; c—significantly different from fasted controls. Summary statistics from independent replicates are in S7 Table. (TIF) [file pgen.1008242.s004.tif]

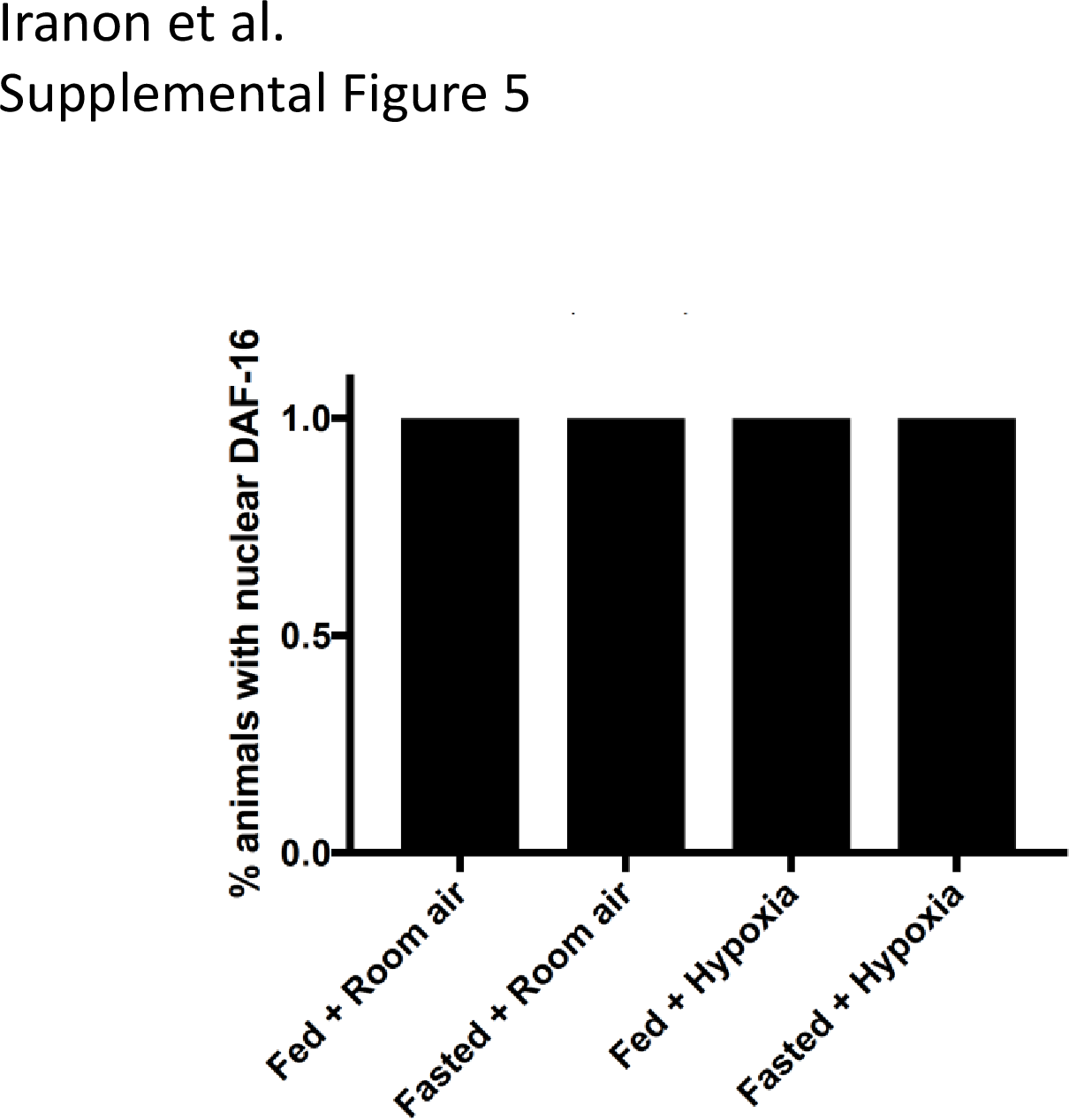

Supplement: S5 Fig — Cohorts of 20 daf-2(e1370) mutants expressing DAF-16::GFP were maintained in room air on food for 24 hours (Fed + Room air), fasted in room air for 24 hours (Fasted + Room air), exposed to hypoxia for 24 hours on food (Fed + Hypoxia), or exposed to hypoxia after fasting (Fasted + Hypoxia; F = 6h; H = 24H). The percent of animals with nuclear GFP was scored immediately post hypoxia. Average data from 3 independent experiments is shown. The bar height indicates the mean. Error bars (present, but not visible) are the standard deviation. (TIF) [file pgen.1008242.s005.tif]

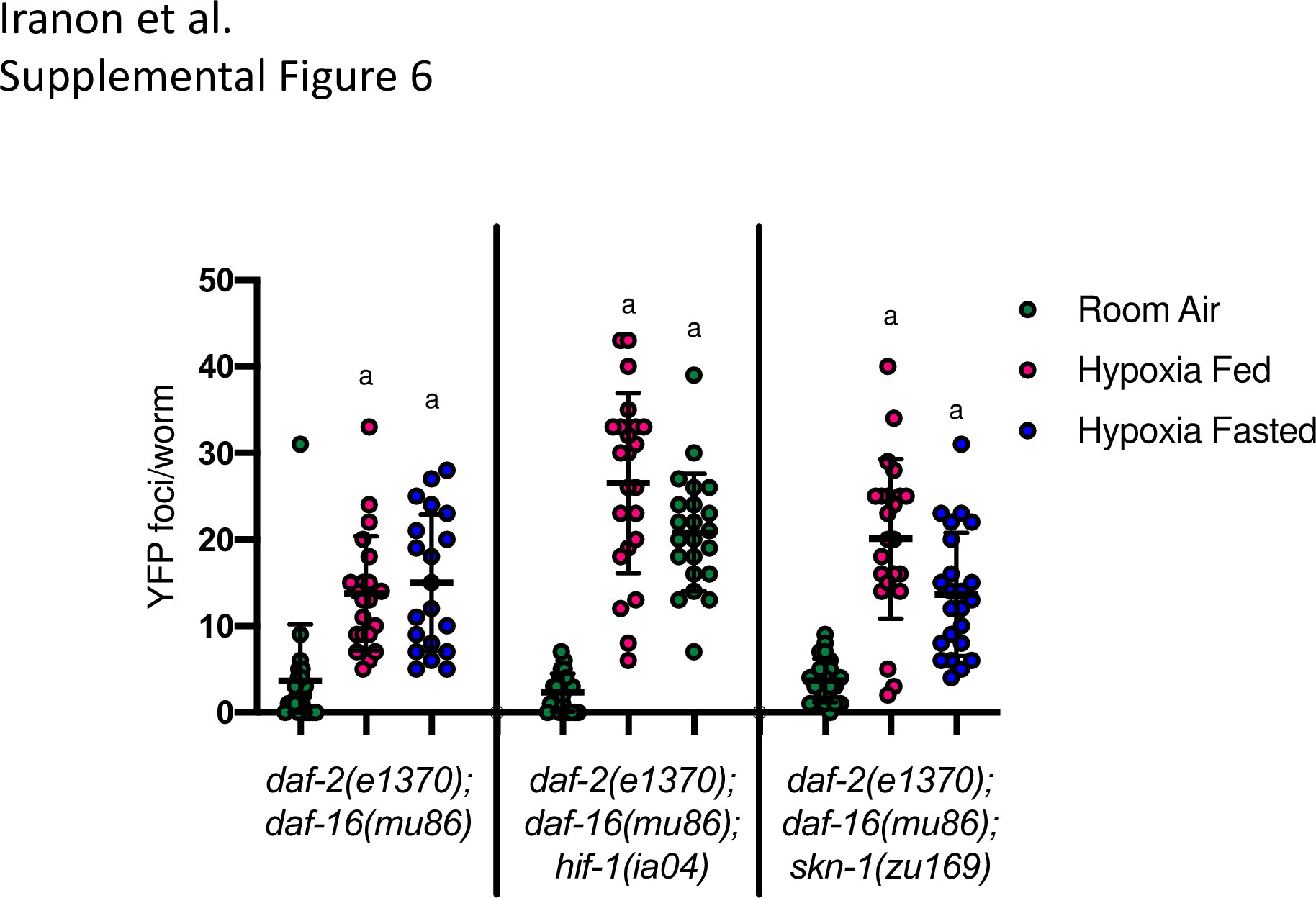

Supplement: S6 Fig — The number of Q35::YFP foci formed after exposure to hypoxia was determined for hif-1(ia04); daf-16(mu86); daf-2(e1370); rmIs132 triple mutant animals, and for skn-1(zu169); daf-16(mu86); daf-2(e1370); rmIs132 triple mutants. Animals were exposed to hypoxia for 24h and the number of YFP foci was scored immediately, as in Fig 5. Data from one representative experiment are shown. Each cohort included at least 20 animals, and each experiment was repeated at least 3 times. Significance was calculated using a Kruskal-Wallis test and Dunn’s multiple comparisons post hoc analysis. Significant differences (p < 0.05) in aggregation for a given strain between conditions are indicated by letters above each group as follows: a—significantly different from room air controls; b—significantly different from fed hypoxic controls; c—significantly different from fasted hypoxic controls. Summary statistics from independent replicates are in S16 Table. (TIF) [file pgen.1008242.s006.tif]

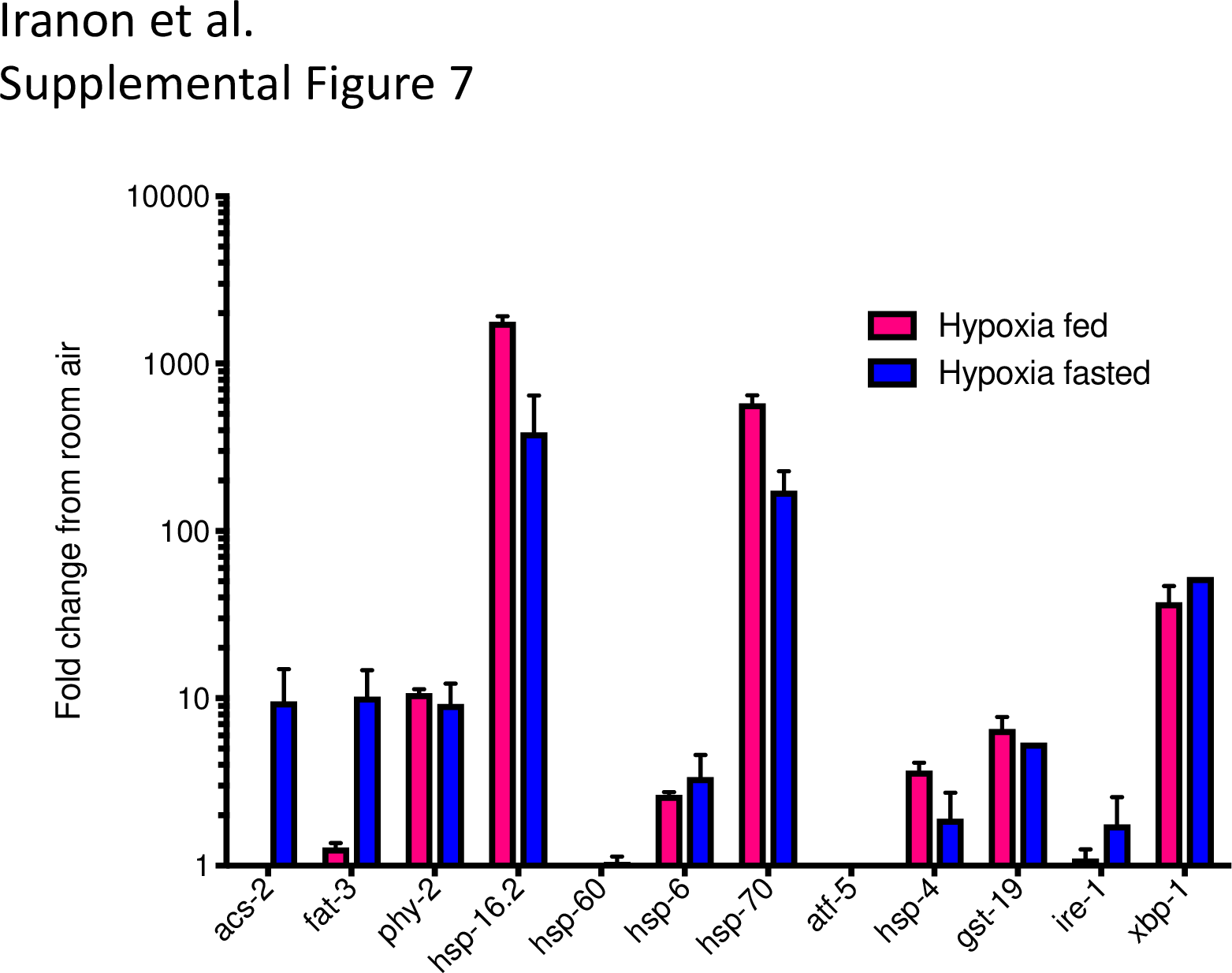

Supplement: S7 Fig — qRT-PCR measurement of mRNA abundance for each gene was measured from three independent replicates. The acs-2 and fat-3 genes are known to by induced by fasting [13]. phy-2 induction in hypoxia is hif-1-dependent [S52]. HSF-1 mediates expression of hsp-16.2, hsp-70, and hsp-4 [S53–55]. There was no statistically-significant difference between expression of any of these genes in animals exposed to hypoxia when fed or fasted. (TIF) [file pgen.1008242.s007.tif]
