# Supplementary material for: Fasting prevents hypoxia-induced defects of proteostasis in C. elegans
Source: PLoS Genet. 2019 Jun 27;15(6):e1008242. doi: 10.1371/journal.pgen.1008242 (PMC6619831; doi:10.1371/journal.pgen.1008242)
Supplement: S10 Table — (DOCX) [file pgen.1008242.s017.docx]

| Strain | Genotype | Reference |
| --- | --- | --- |
| AM140 | *rmls132 [unc-54p::Q35::YFP]* | Saytal et al. 2000 |
| AM141 | *rmls133 [unc-54p::Q40::YFP]* | Saytal et al. 2000 |
| CX51 | *dyn-1 (ky51)* | Clark et al. 1997 |
| CL2006 | *dvls2 [pCL12(unc54/human Aβ peptide 1-42 minigene) + pRF4]* | Link, 1995 |
| CB1370 | *daf-2(e1370)* | Kimura et al. 1997 |
| CF1038 | *daf-16(mu86)* | Lin et al. 1997 |
| TJ356 | *zls356 [daf-16p::daf-16a/b::GFP + rol-6(su1006)]* | Lin et al. 2001 |
| GR1895 | *daf-2(e1370); mgls67 [daf-16p::daf-16::GFP + rol6(su1006)]* | Riedel et al. 2013 |
|  | *daf-2(e1370); Q35::YFP* | * |
|  | *daf-2(e1368); Q35::YFP* | * |
|  | *daf-16(mu86); Q35::YFP* | * |
|  | *daf-2(e1370); daf-16(mu86); YFP::Q35* | * |
| ZG31 | *hif-1(ia04)* | Jiang et al. 2001 |
| EU35 | *skn-1(zu169)/nT1* |  |
| FX30203 | *tmC25[unc-5(tmIs1241 Pmyo-2::Venus)]* | Iwata et al. 2016 |
|  | *hif-1(ia04); daf-2(e1370); daf-16(mu86); YFP::Q35* | * |
|  | *skn-1(zu169)/tmC25[unc-5(tmIs1241 Pmyo-2::Venus)];*  *daf-2(e1370); daf-16(mu86); YFP::Q35* | * |

* Strain was created for this study by crossing AM140 into indicated genetic background. Mutant alleles were verified phenotypically and by using PCR genotyping when appropriate.
