# Supplementary material for: Fasting prevents hypoxia-induced defects of proteostasis in C. elegans
Source: PLoS Genet. 2019 Jun 27;15(6):e1008242. doi: 10.1371/journal.pgen.1008242 (PMC6619831; doi:10.1371/journal.pgen.1008242)
Supplement: S1 Text — (DOCX) [file pgen.1008242.s018.docx]

S18 Text: Supplemental references

S52. Shen C, Nettleton D, Jiang M, Kim SK, Powell-Coffman JA (2005) Roles of the HIF-1 hypoxia-inducible factor during hypoxia response in *Caenorhabditis elegans*. J Biol Chem 280: 20580-20588.

S53. Guisbert E, Czyz DM, Richter K, McMullen PD, Morimoto RI (2013) Identification of a tissue-selective heat shock response regulatory network. PLoS Genet 9: e1003466.

S54. Hajdu-Cronin YM, Chen WJ, Sternberg PW (2004) The L-type cyclin CYL-1 and the heat-shock-factor HSF-1 are required for heat-shock-induced protein expression in *Caenorhabditis elegans*. Genetics 168: 1937-1949.

S55. MacNeil LT, Pons C, Arda HE, Giese GE, Myers CL, Walhout AJ (2015) Transcription Factor Activity Mapping of a Tissue-Specific in vivo Gene Regulatory Network. Cell Syst 1: 152-162.
